# Regionalization of pancreatic surgery in California: Benefits for preventing postoperative deaths and reducing healthcare costs^☆^

**DOI:** 10.1016/j.sopen.2023.11.004

**Published:** 2023-11-20

**Authors:** Lauren M. Perry, Robert J. Canter, Cameron E. Gaskill, Richard J. Bold

**Affiliations:** Division of Surgical Oncology, Department of Surgery, University of California, Davis, Medical Center, Sacramento, United States of America

**Keywords:** Pancreatic surgery, Volume:outcome relationship, Regionalization

## Abstract

**Introduction:**

Pancreatic cancer (PC) surgery has been associated with improved outcomes and value when performed at high-volume centers (HVC; ≥20 surgeries annually) compared to low-volume centers (LVC). Some have used these differences to suggest that regionalization of PC surgery would optimize patient outcomes and expenditures.

**Methods:**

A Markov model was created to evaluate 30-day mortality, 30-day complications, and 30-day costs. The differences in these outcome measures between the current and future states were measured to assess the population-level benefits of regionalization. A sensitivity analysis was performed to evaluate the impact of variations of input variables in the model.

**Results:**

Among 5958 new cases of pancreatic cancer in California in 2021, a total of 2443 cases (41 %) would be resectable; among patients with resectable PC, a total of 977 (40 %) patients would undergo surgery. In aggregate, HVC and LVC 30-day postoperative complications occurred in 364 patients, 30-day mortality in 35 patients, and healthcare costs expended managing complications were $6,120,660. In the predictive model of complete regionalization to only HVC in California, an estimated 29 fewer complications, 17 fewer deaths, and a cost savings of $487,635 per year would occur.

**Conclusions and relevance:**

Pancreatic cancer (PC) surgery has been associated with improved outcomes and value when performed at high-volume centers (HVC; ≥20 surgeries annually) compared to low-volume centers (LVC). Complete regionalization of pancreatic cancer surgery predicted benefits in mortality, complications and cost, though implementing this strategy at a population-level may require investment of resources and redesigning care delivery models.

## Introduction

There has been substantial research over the past two decades demonstrating a strong, consistent association between high-volume surgical centers and improved perioperative outcomes, specifically for operations associated with high mortality, including pancreatectomy [1,2]. Pancreatectomies are relatively uncommon operations and the ability to develop technical expertise is largely limited by experience. As such, only a small number of high-volume surgical centers have emerged for these complex procedures. With a strong relation between procedural volume and clinical outcome as well as the inherent high baseline risks associated with pancreatic resection, pancreatectomy is a common model evaluated for regionalization [3,4]. Based on data demonstrating improved perioperative outcomes after pancreatic resection at high-volume centers, in January 2004 the Leapfrog group coalition of health care purchasers added pancreatic resection to the list of procedures targeted for evidence-based referral in effort to concentrate high-risk surgeries in centers that have the best results [3,[5], [6], [7]]. Since that time, operative volumes at high-volume centers have modestly increased [4,8,9].

However, additional important considerations of regionalization pertain to economic evaluation and specifically the cost-effectiveness of facilitating high-volume care. Some follow-up studies have previously examined the impact of high-volume care on costs and value in pancreatic cancer [[10], [11], [12], [13], [14]]. For example, a recent cost-effectiveness analysis examining pancreatic resections at high-volume centers in California demonstrated a modest survival benefit that was cost-effective by many oncology standards, albeit with increased overall costs [13]. Though high-volume care was cost-effective by the metrics in that analysis, economic outcomes must continually be re-evaluated. In particular, the cost-effectiveness of pancreatic resection regionalization warrants continued analysis given its complexity of care, poor overall survival, and growing incidence. Furthermore, improvements in perioperative care have narrowed the gap in clinical outcomes between high- and low-volume centers, though significant differences still exist [4,15,16]. Currently, pancreatic cancer accounts for the fourth‑leading cause of cancer-related deaths in the United States, but by the year 2030, it is projected to be the second‑leading cause [17]. A growing incidence of pancreatic cancer begs the question of whether regionalization will be a cost-effective strategy in the future and what the true clinical impact will be.

Given the difference in outcomes of pancreatic surgery when patients are cared for a low-volume centers compared to high volume centers, there have been calls to regionalize pancreatic surgery primarily into high-volume centers to achieve optimal population-based outcomes. Despite these calls, current data suggests that approximately 50 % of all pancreatic resections are already performed at high-volume centers, but given geographic as well as other socioeconomic factors, the remainder of the population is cared for at low-volume centers [3,9,18]. The difference in postoperative mortality rate between high-volume centers and low-volume centers suggests that there is the potential to reduce the number of patients who could potentially have their postoperative death prevented if regionalization of pancreatic surgery were implemented. In light of these findings, we sought to estimate the improvement in clinical outcomes and healthcare expenditures, if any, if all pancreatic cancer surgery in California occurred at high-volume centers (i.e. complete regionalization of care). We performed a Markov model analysis comparing the current state of care and healthcare costs to that if all pancreatic cancer surgery were performed at high-volume centers in California. This allowed us to test the hypothesis that complete regionalization would amount to markedly decreased mortality, complications, and costs, thus supporting healthcare policies promoting regionalization.

## Methods

**Fig. 1:**
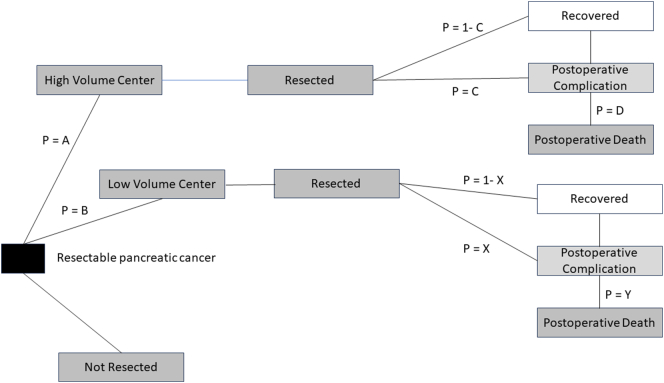
Markov model diagram demonstrating event states of patients with pancreatic cancer. Model variables include the probability of patients undergoing surgery at a HVC (p = A) or LVC (p = B), the incidence of a postoperative complication at a HVC (p = C) or at a LVC (p = X) and the incidence of a postoperative death at a HVC (p = D) or at a LVC (p = Y).

Markov modeling is a method of predicting cost-effectiveness by cycling patients through health states relative to a particular disease whereby they accumulate costs and effects. The Markov model assumptions are dependent on current literature results as input values. At the end of the model, the total cost-effectiveness ratio for the intervention is calculated by summing up all the weighted costs and effects for each cycle; this type of modeling allows evaluation of decisions that result in economic outcomes over a long period of time [19]. For this study, a computer-generated Markov model was created to compare two states – the current state of care and healthcare costs, and the future state. For the current state, patients with resectable pancreatic cancer received care at high- or low-volume surgery centers according to input variables obtained from the literature and had resulting 30-day outcomes (mortality, complications, costs) (Fig. 1). For the future state, all patients with resectable pancreatic cancer received care at high-volume surgery centers and assumed associated 30-day outcomes (mortality, complications, costs). The 30-day outcomes between the current and future states were compared to assess the cost-effectiveness of complete regionalization of pancreatic resections in California. Although there is increasing evidence that the risk of postoperative mortality persists past 30 days and there has been recent suggestion that 90-day perioperative mortality may be a better measure, our study used 30-day outcomes given the more robust data for selection of the input variables in the model. Our literature search strategy started with the comprehensive literature review of pancreatic cancer outcomes based on hospital volume by Acher et al. in 2020 as well as a recent meta-analysis by Fischer et al. [20,21] We then performed an additional PubMed literature search for the past three years using the search criteria: ((pancreas or pancreatic) AND (cancer) AND (regionalization OR hospital OR volume-outcome) AND (morbidity OR complications OR mortality OR costs)). Original research studies were selected for critical review and inclusion in Table 1. Given that some input variables were noted to have significant ranges, four a priori strategies were utilized to select input variables: 1) when ranges existed from the same dataset, the input variable for the initial model was in the middle of the range, 2) datasets that focused on outcomes in California were chosen as the model was developed around that cohort of patients, 3) input variables were cohorted when the same dataset was used for different variables, and 4) multi-institutional datasets were selected over single institutional datasets. In the sensitivity analysis, input variables were modified within the ranges or variables identified in the literature search.

**Table 1:** Published data referenced in selection of model input variables.

Input Variables	Published Data	Data Source	Timeframe	Selected Value	References
Proportion of patients treated across center types (in California)	53.4 % HVCs 46.6 % LVCs	CCR	2004–2012	53.4 % 46.6 %	Perry et al. [13]
Projected 2021 incidence of pancreatic cancer in California	5958	SEER	1975–2017	5958	Siegel et al. [22]
Proportion of patients presenting with localized or regional disease	41 %	SEER	1975–2017	41 %	Siegel et al. [22]
Proportion of eligible patients undergoing pancreatic resection	21–41 %	SEER NCDB	1999–2004 1995–2004 2004–2011	40 %	Riall et al. [18] Riall et al. [23] Bilimoria et al. [24] Shapiro et al. [25]
30-day mortality rates	1.7–2.1 % HVCs 4.5–6.1 % LVCs	NCDB	2004–2015 2004–2013	1.8 % HVCs 5.2 % LVCs	Panni et al. [15] Jogerst et al. [16]
30-day complication rates	37.2 % overall 34.3 % HVCs 39.8 % LVCs	CCR	2004–2012	34.3 % HVCs 39.8 % LVCs	Perry et al. [13]
	37.9 % 33.7 % HVCs 41.3 % LVCs	NIS	2002–2011		Gani et al. [26]
Mean 30-day hospitalization costs	$62,561 HVCs 59,525 LVCs	Vizient	2004–2012		Bateni et al. [11]
	$30,395 HVCs $29,048 LVCs	NIS	2002–2011		Gani et al. [26]
Cost of Complication	$16,815	CCR/OSHPD	2004–2012	$16,815/complication	Perry et al. [13]
	$17,947	NIS	2002–2011		Gani et al. [26]
	$9101	Single Institution	2010–2017		Jaija et al. [27]
	$11,682	NIS	2004–2017		Alteiro et al. [28]

Abbreviations: HVCs, high-volume centers; LVCs, low-volume centers; CCR, California Cancer Registry; SEER, Surveillance, Epidemiology, and End Results Program; NCDB, National Cancer Database.

The model assumes that the proportion of patients receiving care at high- and low-volume centers is consistent with our prior study and that pancreatic resections were nearly evenly split between high-volume (46.6 %) and low-volume centers (53.4 %) [13]. Input variables for the model were obtained from the literature and included the following: 1) the projected incidence of pancreatic cancer in California for the year 2021; 2) the percentage of newly-diagnosed patients presenting with localized or regional disease and thus eligible for resection; 3) the percentage of patients eligible for resection who underwent surgery; 4) 30-day mortality rates; 5) 30-day complication rates; and 6) mean 30-day costs [11,13,15,16,18,[22], [23], [24], [25]]. For this model, high-volume centers were defined as centers performing at least 20 pancreatic resections annually based on our prior research and literature showing improved mortality at this cutoff [13,[29], [30], [31]]. Although there have been other breakpoints utilized in the separation of low-volume centers from high-volume centers, and improvements in postoperative outcomes have been noted to further improve in “super-high volume centers”, we chose the breakpoint of 20 resections per year based on the relative frequency of this definition in the published literature. Additional details of the model input variables are outlined in Table 1.

Estimates for 30-day mortality rates were based on two recent National Cancer Database Analyses each comparing postoperative mortality rates for high-volume and low-volume centers after pancreatic resection. In these studies, high-volume 30-day mortality rates ranged from 1.7 to 2.1 %, while low-volume 30-day mortality rates ranged from 4.5 to 6.1 % [15,16]. Ultimately, the averages of each mortality range were calculated, and the final 30-day mortality rates chosen for the model were 1.8 % and 5.2 % for high- and low-volume centers, respectively.

Estimates for 30-day complication rates for high- and low-volume centers were obtained from our prior cost-effectiveness analysis utilizing the California Cancer Registry linked to the Office of Statewide Health Planning and Development [13]. For that study, the overall 30-day complication rate was 37.2 %. On subgroup analysis stratifying by volume status, the 30-day complication rates were 34.3 % and 39.8 % for high- and low-volume centers, respectively. These complication rates were included in the in present model.

Cost data were derived from a prior University HealthSystems Consortium database (Vizient) database analysis in which Bateni et al [11] suggested that postoperative complications are the primary driver of pancreatic resection hospitalization costs rather than surgical volume itself. Specifically, the authors did not detect any difference in mean costs for pancreatic resections performed at high- and low-volume centers but did identify that each postoperative complication was associated with a mean cost of $16,815. Thus, a $16,815 cost per postoperative complication was applied to our model in both the current and future states given the knowledge that postoperative complications are a major source of healthcare expenditures. Furthermore, our model assumed that the cost of the initial pancreatic resection was the same at either a high or low-volume center, and thus the primary difference in overall perioperative costs was related to the management of complications [11]. As previously published, these costs were estimated from the summation of individual itemized charges for the hospitalization in which the pancreatic resection occurred and then multiple by hospital revenue code specific cost-to-charge ratios and adjusted for geographic variation with wage indices. These costs were also adjusted for inflation to 2016 U.S. dollars [11,32].

Approval by the UC Davis Institutional Review Board was not required as there are no human subjects in the current research.

## Results

**Fig. 2:**
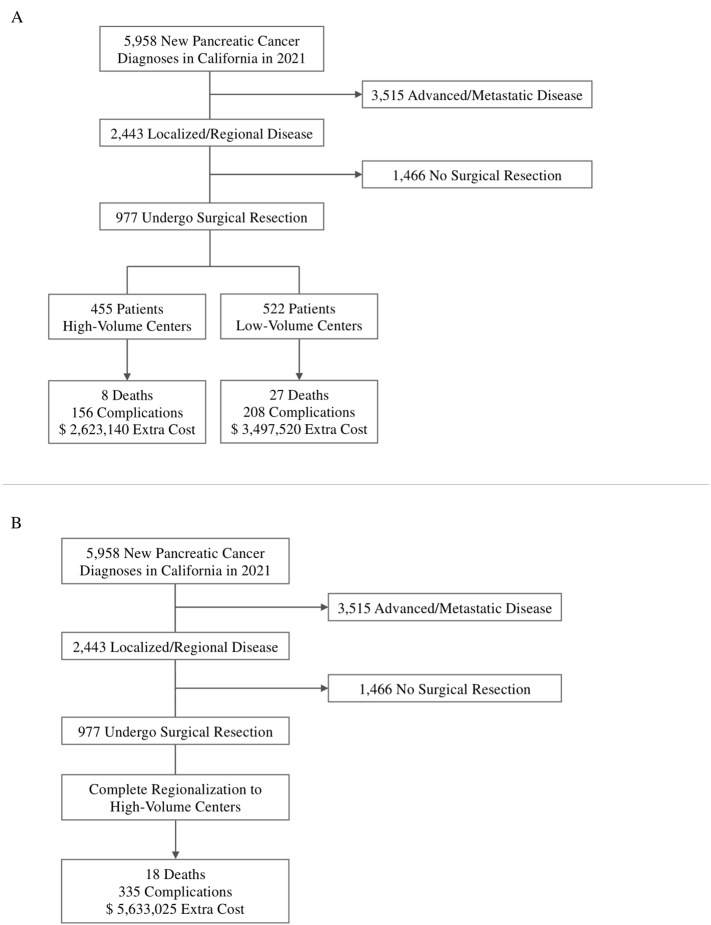
Cohort Selection and Outcomes for the Current State (A) and Future State (B) Models.

There were an estimated 60,430 new cases of pancreatic cancer in the United States in 2021, with California accounting for 5958 of those [20]. Of these new cases, approximately 41 % were expected to be localized or regional disease that would be eligible for curative-intent resection. Among these patients who were eligible for resection, others have observed that only 27–41 % will undergo pancreatic resection due to a variety of barriers, including treatment biases and comorbid diseases [18,[23], [24], [25]]. For our model, we made the optimistic assumption that 40 % of eligible patients would ultimately undergo resection for pancreatic. Cohort selection and outcomes are outlined in Fig. 2.

Therefore, for the input variables of our model, we estimated that of 5958 new cases of pancreatic cancer in California in 2021, a total of 2443 cases (41 %) would be resectable; among patients with resectable pancreatic cancer, a total of 977 (40 %) patients would undergo surgery. For the current state of the model, surgeries were nearly even split between low and high-volume centers based on our prior analysis of healthcare delivery patterns, with 522 (53.4 %) cases performed at low-volume centers and 455 (46.6 %) at high-volume centers. In the current state model, the 522 pancreatic resections performed at low-volume centers were predicted to result in 27 deaths and 208 complications within 30 days. These 30-day complications would cost low-volume centers an extra $3,497,520. The 455 resections performed at high-volume centers were predicted to result in 8 deaths and 156 complications within 30 days. These 30-day complications would cost high-volume centers an extra $2,623,140. In aggregate for low- and high-volume centers, the model predicted that in 2021, there would be 35 deaths and 364 complications within 30 days after pancreatic cancer surgeries. These 30-day complications would amount to an extra cost of $6,120,660 beyond the cost of the initial pancreatic resection.

For the future state model, all patients with resectable pancreatic cancer who underwent surgery received care at high-volume centers. When a total of 977 (100 %) patients underwent surgery at high-volume centers, the model predicted that 18 deaths and 335 complications would result within 30 days of surgery. These 30-day complications would cost high-volume centers an extra $5,633,025. When comparing the future state where all resections occur at high-volume centers to the current state, the future state resulted in 17 fewer deaths and 29 fewer complications within 30 days of surgeries. These fewer complications would result in a cost savings of $487,635. All model results are shown in Table 2.

**Table 2:** Markov model outputs predicting 30-day mortality, complications, and costs for pancreatic resection regionalization.

Model 1 – Current State	Surgeries	Deaths	Complications	Complication Costs^a^, $
Low-volume centers	522	27	208	$3,497,520
High-volume centers	455	8	156	$2,623,140
Total	977	35	364	$6,120,660
Model 2 – Future State	977	18	335	$5,633,025
Modeling Differential		−17	−29	-$487,635

a Costs are directly attributable to the number of complications, $16,815/complication [11].

We then tested the model for sensitivity related to our assumptions based on published data from national administrative databases. We evaluated the impact of incomplete regionalization of pancreatic surgery by changing the assumption that only 75 % of operations would be performed in high volume centers from a baseline of 46.6 %. In this model, the future state resulted in 9 fewer deaths and 15 fewer complications with a cost savings of $256,870; all outcomes of lesser impact than complete regionalization. We also tested the financial impact of regionalization by altering the healthcare cost for management of complications as our assumption was that costs of managing complications were the same in HVC compared to LVC. Recognizing that high volume centers have lower rates of complications, we postulated that the management of complications in high volume centers may also be more efficient and therefore associated with lower costs and created a model in which the cost per postoperative complication was double at a LVC compared to a HVC ($22,364 and $11,098 respectively). In this model, the cost savings dramatically increased to $2659, 211. Finally, we evaluated the impact of widening the range of complications between HVC and LVC to 31.5 % and 43 % respectively, which would create a 30 % difference in the incidence of postoperative events between HVCs and LVCs. In this model, the future state resulted in 58 fewer complications with a cost savings of $974,295.

## Discussion

Our modeling of complete regionalization of surgical care for pancreatic cancer to high-volume centers in California yielded 17 lives saved and averted 29 complications for a global cost savings of just under $500,000. These results were far smaller than the anticipated impact of complete regionalization of pancreatic resection but can be explained by several observations. The first is that both the incidence and mortality gap between high-volume and low-volume centers for clinical outcomes has narrowed significantly over the past 20 years. In the original publication regarding operative mortality rates and hospital volume by Birkmeyer et al [1], the mortality rate for pancreatectomy was 15.4 % at low-volume centers and 3.8 % at high-volume centers. However, recent reports and our current model assumptions demonstrate significant overall mortality improvements and narrowing of this treatment gap between low-volume and high-volume centers to approximately 5 % and 2 %, respectively [15,16]. Similarly, our results demonstrated a 50 % relative risk reduction in 30-day mortality when comparing our future and current state Markov models (18 and 35 deaths, respectively). While this relative risk reduction supports existing knowledge that high-volume care decreases mortality for pancreatic resection, the absolute reduction was still far smaller than anticipated, especially when considering population-level policies. A second potential explanation for the marginal differential predicted by the model is that the absolute number of patients undergoing curative-intent pancreatic resection is lower than conceptually anticipated. Various population-based analyses have observed that only 27–41 % of patient with potentially resectable disease will undergo surgical resection due to possible treatment barriers such as geography, socioeconomics, and patient and physician biases that limit surgical access [18,[21], [22], [23]]. However, whether regionalization would increase the delivery of surgical care to patients with resectable disease is unclear, since many of the same treatment barriers would still exist.

In our sensitivity analysis of modifying three input variables (degree of regionalization, incidence of complications, and cost of complications), the greatest impact was seen on the cost of complications if HVCs were able to manage the postoperative complication is more cost effective manner. However there are conflicting reports of the cost of care at HVCs compared to LVCs including a study we have previously published that indicates that the cost of post-surgical care at HVCs may in fact be higher than at LVCs [13]. If this is the case, then the economic consequence of regionalization may in fact be blunted as the lower risk of complications at HVCs would be offset by the higher cost when patients are managed at HVCs compared to LVCs. Perhaps contributing to this is a recognized characteristic of HVCs in effectively managing the index operation with improved outcomes and lower costs, but also the postoperative events. The impact of surgical care in LVCs may not be on the overall incidence of postoperative complications, but the failure to rescue leading to more severe and costly complications.

The volume-outcome relationship in complex operations has been well-documented for over twenty years and has served as the impetus for regionalization recommendations of low-volume, high-risk procedures [[1], [2], [3], [4], [5], [6], [7],^9^,^21^]. While there have been some efforts to foster regionalization of these procedures such as the Leapfrog Group recommendations and outreach from high-volume academic centers, healthcare payors have traditionally not dictated care through covered benefits or network guidelines. While our model did predict that complete regionalization would have measurable benefits, these anticipated benefits were modest and likely to be significantly offset by barriers in care coordination. As an example, Riall et al [3] observed a <10 % increase in pancreatic resections at high-volume centers in Texas during a five-year period (1999–2004), while >25 % of patients were still treated at very low-volume centers (<5 resections/year) at the end of the study period. These trends were attributed to significant residual barriers to regionalization, including patient inconvenience, physician referral patterns, health system alignments, and geographic impact, which highlights the important point that a high-volume referral may not be a reasonable option for some patients. Therefore, the benefits of regionalization must be weighed against their potential detriments to patients, which include logistical inconvenience (increased travel time, expenses, and time off work) and reduced access to qualified surgical care potentially present at low-volume centers [33,34]. The potential detriments of regionalization to the healthcare system must also be considered, which include the potential for overwhelming high-volume centers and further increasing the perioperative outcomes gap between high- and low-volume centers as a result of further limiting exposure to these complex surgical cases [3,33]. Despite persistent barriers and the potential detriments of regionalization, ongoing efforts to promote regionalization continue under the assumption that overall population-level outcomes will be superior if all care is delivered in high-volume centers [20]. In fact, this was the justification of the development of trauma systems over four decades ago. In a critical analysis of patient outcomes for severe trauma, communities without regionalization of trauma systems (Orange County, CA) compared to a community with regionalization of trauma care (San Francisco, CA), at least 28 % of the deaths observed in a non-regionalized system were considered preventable if care had been provided in a dedicated trauma center which was specifically developed to regionalize care of the severely injured patient [35]. At a policy level, this effort of regionalization of trauma care was based on the conceptual model of alignment of critical resources for optimal patient care and outcomes [36]. The exact impact of reduction of trauma-related mortality remains uncertain, the 17 fewer deaths per year for the entire pancreatic surgical volume of California seems to have less overall community benefit likely related to the difference in incidence of the two “diseases”.

In weighing the benefits of pancreatic resection regionalization, it is essential to also consider its unintended consequences, specifically who may be managing the postoperative care of these complex surgical patients. In regions with significant geographic dispersion of the population, limited access for urgent medical care has been well-recognized [37,38]. If more patients have a long travel distance for their complex surgeries, this can create barriers to the provision of timely and appropriate after care. This is of particular importance when considering pancreatic cancer, as the incidence of 30-day readmissions following resection is approximately 15–20 % [11,13,39]. Although the proposed benefits of regionalization to high-volume centers include lower complication and readmission rates [40], these events would realistically remain a frequent occurrence and community hospitals may be the front-line for many patients initially presenting with postoperative complications after surgeries that were performed at high-volume centers. There is significant evidence that patients with critical illnesses presenting for evaluation in rural emergency departments that require transfer for a higher level of care have worse outcomes than those undergoing initial evaluation in comprehensive emergency departments [1,41,42]. Therefore, there may be a tradeoff between immediate improved perioperative outcomes at high-volume centers and the deleterious effect of poorer outcomes resulting from delayed management or inability to transfer. While the short-term benefits of regionalization are enticing, the downstream effects on community healthcare systems and processes of care must be carefully weighed to ensure that care is high quality yet still accessible to patients throughout all phases of surgery.

## Limitations

This study has some important limitations inherent to the modeling analyses. First, the model inputs and assumptions are estimates based on the best available literature available for reference. Model estimates must be considered within context and for this reason our model outputs are only compared to one another and not to external data. We hypothesize that the model results may differ depending on the threshold chosen for HVC determination (e.g., 12, 20, or 30 pancreatic resections annually). Given variability in the literature regarding the definition of HVCs, we chose a moderate HVC threshold (≥ 20 cases/year) for the modeling analysis and acknowledge that more extreme thresholds could be chosen depending on the research question. Additionally, we chose a high pancreatic resection rate for our model and acknowledge that selecting a lower resection rate would slightly blunt the effects of regionalization, while selecting an even higher rate would further pronounce the model findings. Second, Markov modeling is a method of predicting outcomes such as costs and effects at a population level in order to inform healthcare reform or policy. The aim of our study was to assess the cost-effectiveness of receiving high-volume care for a very narrow cohort in order to better understand the perioperative outcomes and economic ramifications of potential policy-level decisions. We studied a sample of patients with early-stage pancreatic cancer in California who underwent surgery; analysis of this retrospective cohort does not allow broad causal conclusions about high- or low-volume care in general. For example, there may be further aspects of care not included in our Markov model such as optimal preoperative imaging, improved multidisciplinary care, adherence to guideline-compliant care, and provision of support services for which care at high-volume centers may improve patient outcomes compared to low-volume centers so that the benefit is greater than our model suggested from regionalization of pancreatic cancer surgery. Regionalization of the treatment decision making may lead to more patients undergoing surgery for resectable pancreatic cancer and this potential benefit was also not included in our model [43].

## Conclusions

In this predictive Markov modeling analysis, we demonstrated that complete regionalization of pancreatic resections to high-volume centers in California would result in an estimated 17 fewer deaths and 29 fewer complications within 30 days of surgery, and a cost savings of nearly $500,000 per year. While complete regionalization predicted benefits in mortality, complications, and cost, these benefits at a population-level were far smaller than anticipated and the operational challenges of care coordination would be significant. These data suggest that policy efforts around regionalization of care based on volume:outcome relationships should include the incidence of the disease at a population level, the exact gap between high- and low-volume providers and the tradeoff of resource investment to cost savings. Our data suggest that for pancreatic diseases, policies directed at complete regionalization of surgery may not yield significant community benefits, Instead, ocus should be placed on mitigating barriers to surgical care, including addressing geographic disparities, patient- and physician-level biases of the disease, and broadening networks for care delivery to continue to narrow the gap between high- and low-volume centers.

## CRediT authorship contribution statement

Drs Perry and Bold collected the data, performed the analysis and created the first draft of the manuscript. Drs Bold, Gaskill and Canter provided critical editing and final approval of the manuscript.

## Funding/support statement

No extramural funding was received for the conduct of this research.

## Ethics approval

Approval by the UC Davis Institutional Review Board was not required as there are no human subjects in the current research.

## Declaration of competing interest

The authors have no related conflicts of interest to declare.
